# Implications of Twitter in Health-Related Research: A Landscape Analysis of the Scientific Literature

**DOI:** 10.3389/fpubh.2021.654481

**Published:** 2021-07-09

**Authors:** Andy Wai Kan Yeung, Maria Kletecka-Pulker, Fabian Eibensteiner, Petra Plunger, Sabine Völkl-Kernstock, Harald Willschke, Atanas G. Atanasov

**Affiliations:** ^1^Oral and Maxillofacial Radiology, Applied Oral Sciences and Community Dental Care, Faculty of Dentistry, The University of Hong Kong, Hong Kong, China; ^2^Ludwig Boltzmann Institute for Digital Health and Patient Safety, Medical University of Vienna, Vienna, Austria; ^3^Institute for Ethics and Law in Medicine, University of Vienna, Vienna, Austria; ^4^Division of Pediatric Nephrology and Gastroenterology, Department of Pediatrics and Adolescent Medicine, Comprehensive Center for Pediatrics, Medical University of Vienna, Vienna, Austria; ^5^Department of Anaesthesia, Intensive Care Medicine and Pain Medicine, Medical University Vienna, Vienna, Austria; ^6^Institute of Genetics and Animal Biotechnology of the Polish Academy of Sciences, Magdalenka, Poland; ^7^Institute of Neurobiology, Bulgarian Academy of Sciences, Sofia, Bulgaria; ^8^Department of Pharmaceutical Sciences, University of Vienna, Vienna, Austria

**Keywords:** health, social media, bibliometric, dissemination, knowledge exchange, twitter

## Abstract

**Background:** Twitter, representing a big social media network, is broadly used for the communication of health-related information. In this work, we aimed to identify and analyze the scientific literature on Twitter use in context of health by utilizing a bibliometric approach, in order to obtain quantitative information on dominant research topics, trending themes, key publications, scientific institutions, and prolific researchers who contributed to this scientific area.

**Methods:** Web of Science electronic database was searched to identify relevant papers on Twitter and health. Basic bibliographic data was obtained utilizing the “Analyze” function of the database. Full records and cited references were exported to VOSviewer, a dedicated bibliometric software, for further analysis. A term map and a keyword map were synthesized to visualize recurring words within titles, abstracts and keywords.

**Results:** The analysis was based on the data from 2,582 papers. The first papers were published in 2009, and the publication count increased rapidly since 2015. Original articles and reviews were published in a ratio of 10.6:1. The Journal of Medical Internet Research was the top journal, and the United States had contributions to over half (52%) of these publications, being the home-country of eight of the top ten most productive institutions. Keyword analysis identified six topically defined clusters, with professional education in healthcare being the top theme cluster (consisting of 66 keywords). The identified papers often investigated Twitter together with other social media, such as YouTube and Facebook.

**Conclusions:** A great diversity of themes was found in the identified papers, including: professional education in healthcare, big data and sentiment analysis, social marketing and substance use, physical and emotional well-being of young adults, and public health and health communication. Our quantitative analysis outlines Twitter as both, an increasingly popular data source, and a highly versatile tool for health-related research.

## Introduction

**Figure 1:**
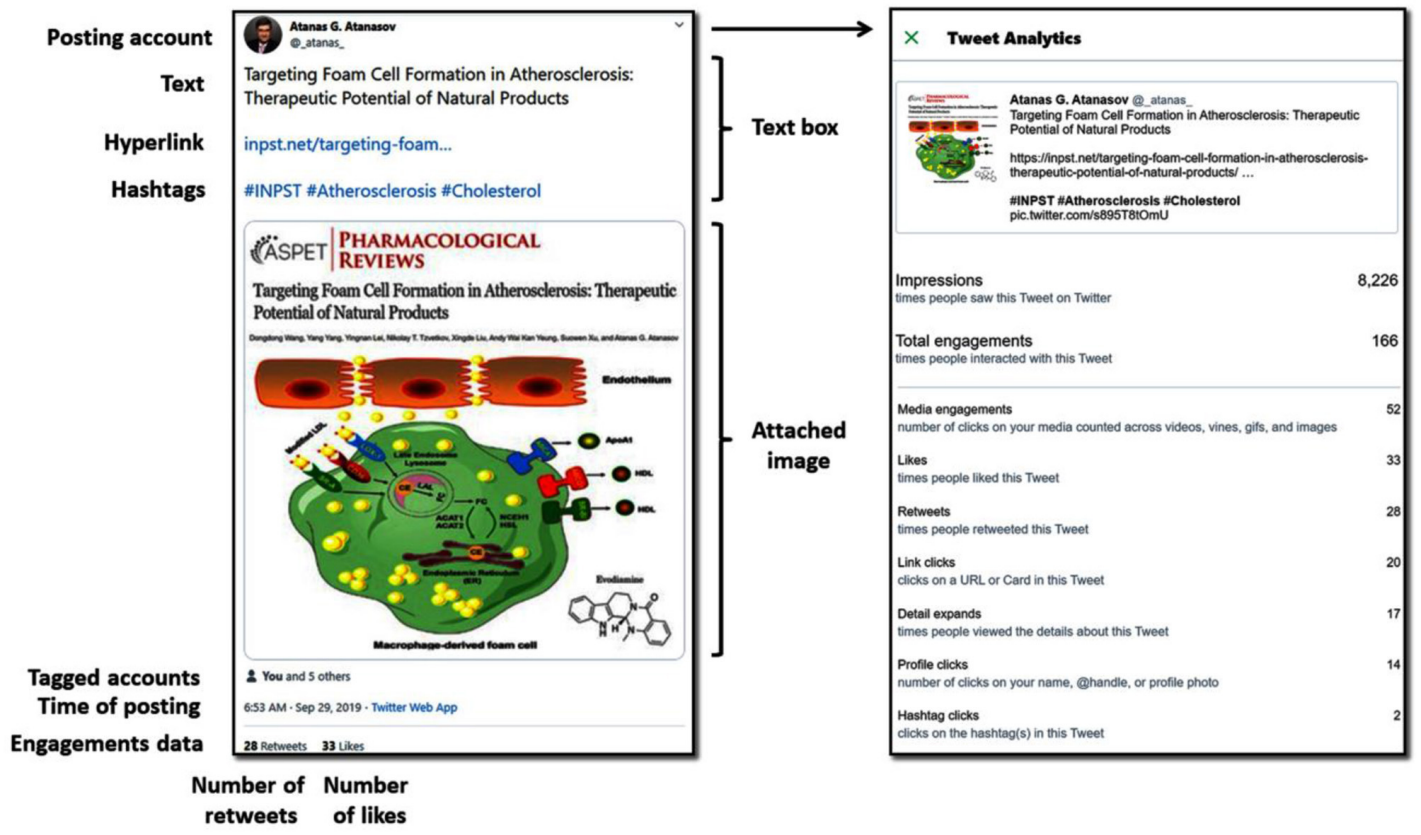
Structure of a typical tweet with text, hyperlink, hashtags, attached image, and tweet analytics. The explanatory scheme is featuring a representative tweet by one of the authors (Atanas G. Atanasov), Available at: https://twitter.com/_atanas_/status/1178170792686886912.

Paralleling the broader access to internet and the increasing smartphone use, social media has developed into a major way of communication for the general population worldwide (1–3). Twitter is one of the most used social media platforms (4). It enables the public distribution of short messages limited to 280 characters (historically, the better-known previous limit was 140 characters). These short messages, termed “tweets,” are usually publicly visible with the exception of limitations by its distributor, to for example a group of approved subscribers, termed “followers.” In addition, tweets can be extended by the attachment of images, videos, specialized hyperlinked keywords termed “hashtags,” and hyperlinks. The structure of a representative tweet is presented in Figure 1. Visibility parameters associated with each tweet include impressions (number of times a user is exposed to a tweet in timeline or search results), total engagements (number of times a user interacted with the tweet), media engagements (number of clicks on attached media such as images/videos), positive appraisal (=likes), sharing (=retweeting), link clicks, detail expands, profile clicks, hashtag clicks (Figure 1), and replies.

Twitter is a platform widely used by scientists and health care professionals for the dissemination of biomedical scientific information. It is the major social media platform contributing to non-traditional visibility metrics such as Altmetrics scores of scientific publications (5, 6). Research distribution via Twitter may aid the general public to access scientific content beyond pay walls and without the need to navigate in complex scientific journal websites. Moreover, it may also aid to clarifying scientific research in lay-men terms, since Twitter only provides a limited amount of characters for each tweet, thus forcing researchers using Twitter to present their results in a more focused and clear way. However, on Twitter health-related statements and personal opinions are also widely disseminated by users without appropriate qualifications, which has contributed to Twitter and other social media networks being major sources of misinformation (7, 8). The latter poses a serious threat to public health, since the internet and social media are rapidly becoming widely-adopted sources of health-related information for the general public (9–11). Aside of being a platform for communication of health-related information, Twitter can be used as a tool for health-related research. A study conducted in 2017 analyzed the different uses of Twitter in 137 health research-related publications. The major uses of Twitter were content analysis (used in 56% of the studies), surveillance (26%), engagement (14%), intervention (7%), recruitment (7%), and network analysis (4%) (12). Moreover, social media networks are not only used as a tool for health research or communication of health-related information, but also might represent independent factors influencing the health of users, with particularly strong impact on mental health aspects such as self-esteem and psychosocial well-being (13). Underling the significance of this platform for the scientific community as a whole, Twitter was identified as the most frequently used professionally social media platform by scientists (14). Taking together the diverse above-described implications and uses of Twitter in bio-medical research, motivated us to focus the current bibliometric study on implications of Twitter in health-related research.

Bibliometric analysis represents a powerful tool for quantitative evaluation of diverse parameters associated with the scientific literature published in a specific area, revealing insights on prevalent research topics, development trends, key researchers, recent publications and scientific institutions (15–18). Briefly, bibliometrics is an umbrella term to cover techniques that track objective metrics of scholarly activities, e.g., publication and citation counts (19). These metrics are also associated with other parameters, such as authors, journals, and publication content (19). Since no bibliometric analysis of Twitter use in the context of health-related research has been conducted so far, we aimed to identify and quantitatively analyze the respective body of scientific literature to gain new insights into this innovative biomedical research area. Relaying on such analysis approach, our objective was to add new layers of information to the existing knowledge by addressing the question how the publication and citation data are relating to contributors in various levels (authors, institutions, countries, etc.) as well as to semantic content. The contributions of this work also aimed to aid the research audience to identify potential collaboration partners, promising research directions, or suitable journals for publishing relevant research findings. Moreover, obtained quantitative data can be of value for a rapid overview of the literature landscape within this research area, which might be useful for both experts and readers from other scientific areas.

## Methods

In March 2020, we searched the electronic Web of Science (WoS) Core Collection database with the following string: (twitter OR tweet^*^ NOT “tweetable abstract^*^”) AND (health^*^ OR medicine^*^ OR illness^*^ OR disease^*^). The search strategy identified papers with these words or derivatives mentioned in their title, abstract or keywords. All papers resulted from the search were preliminarily included. Next, the phrase “tweetable abstract” was added as an exclusion criterion because a preliminary search identified 205 papers published in British Journal of Obstetrics and Gynecology, which requires authors to include a short paragraph of “tweetable abstract” in the abstract section (abstract part suitable to be posted and promoted on Twitter). The Guide for Authors of British Journal of Obstetrics and Gynecology defines “Tweetable abstract” as one part of the abstract that succinctly summarizes the paper (in 110 characters). These papers did not investigate on Twitter use and thus were excluded. No other exclusion criterion was set. No filter was set on publication date. This algorithm, after the exclusion of the mentioned 205 publications from the British Journal of Obstetrics and Gynecology, yielded a final set of 2,582 papers that were further analyzed in this study.

## Data Analysis

The basic bibliographic data of the resultant papers were recorded by the “Analyze” function of WoS. This function enabled us to analyse the frequencies of contributors in terms of authors, institutions, and countries/regions. We then computed citations per paper (CPP) via the “Create Citation Report” for selected subgroups (e.g., for specific authors). The full record and cited references of the identified literature were then exported to VOSviewer for further bibliometric analyses, such as relating citation data to semantic content of the papers and visualization of the results as term maps. A term map was generated to visualize the terms that appeared in the respective titles and abstracts. The terms were identified by VOSviewer using an automatic term identification approach comprised of three steps as described by Van Eck et al. (20). We refrained from manual aggregation since it might involve subjectivity bias (e.g., should Twitter/Tweet/Tweets/tweeting be always merged?). Due to the analysis resulting in massive amounts of keywords, only the most abundant terms (appearance in at least 1% of the analyzed literature; *n* = 26) were included for further analysis. A keyword map was generated analogous for author keywords that appeared in at least 5 papers.

## Results

**Figure 2:**
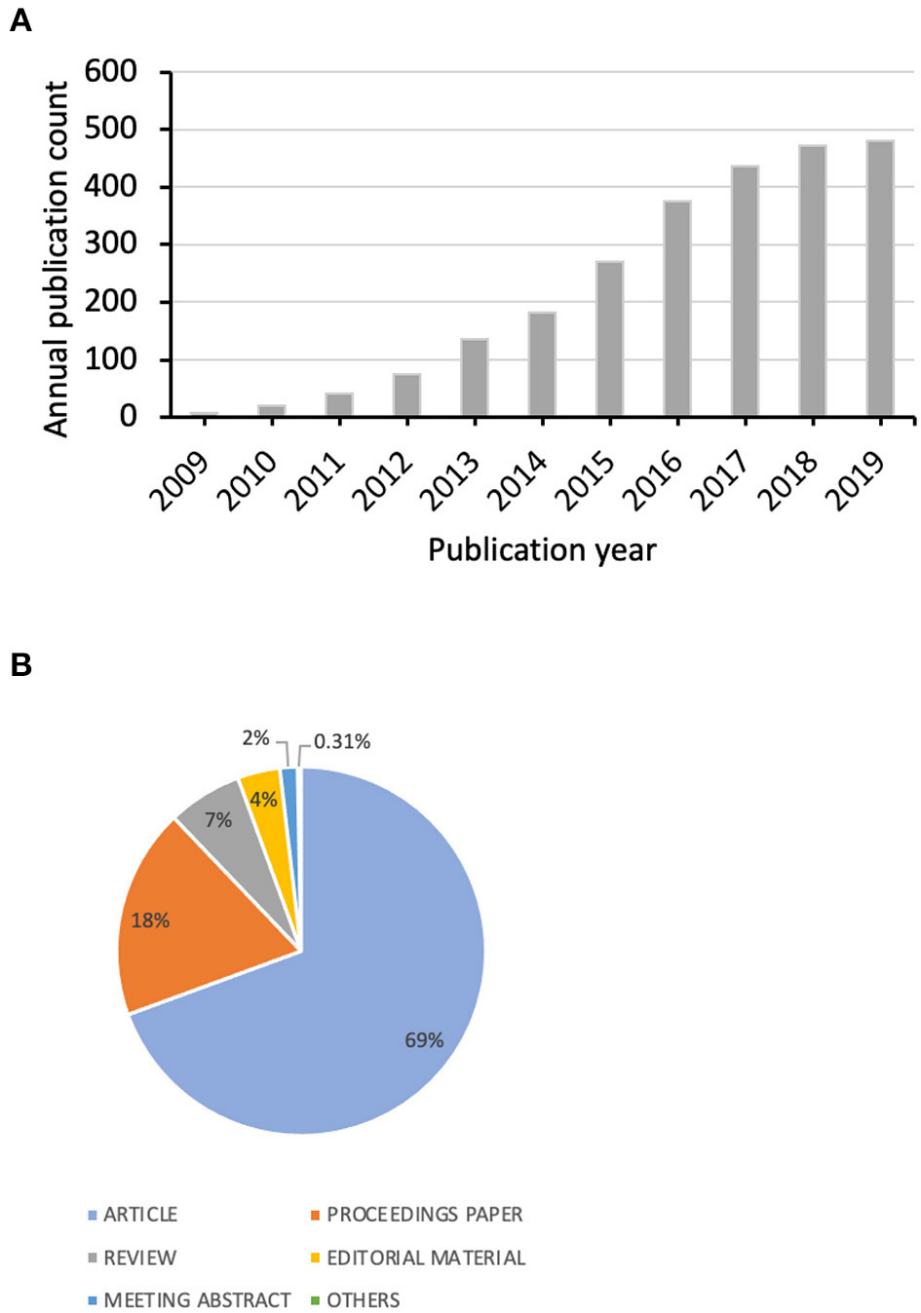
**(A)** Annual publication count of research papers concerning Twitter and health. **(B)** Paper counts by publication type.

The analysis was based on the data from 2,582 papers that were identified with the applied search string. The first papers were published in 2009, and the publication count increased rapidly since 2015 (Figure 2). Total publication counts exceeded 1,000 in 2016 and 2000 in 2018. Original articles and reviews accounted for three-fourths of total publications, in a ratio of 10.6:1 (1,792 vs. 169). Proceedings papers and editorial materials accounted for another 18.4% and 3.7% of total publications (Figure 2B). Over 97% of the publications were written in English. The most cited among the 2,582 analyzed papers was written by Boyd and Crawford and represents an opinion article about the cultural, technological and scholarly aspects of big data usage including social media interaction data from Twitter (21).

The top ten most productive (by number of publications) authors, institutions, countries, journals and Web of Science categories are listed in Table 1. The most productive author was Dr. John S. Brownstein from Harvard University. He published 22 papers in this research area, two of which with over 100 citations, which utilized social and news media contents, including relevant tweets, to predict disease activity and outbreak characteristics for cholera in Haiti, and for influenza in the United States (22, 23). Altogether, the United States had contributions to over half (52%) of the publications, and eight of the top ten most productive institutions were based in this country. The papers were mostly published in journals belonging to the categories public environmental occupational health, health care sciences services, computer science information systems, and medical informatics.

**Table 1:** Top ten most productive authors, institutions, countries, journals and web of science categories.

	**Publication count**	**Citations per paper (CPP)**
**Author**		
John S. Brownstein	22 (0.9%)	27.2
Raina M. Merchant	18 (0.7%)	23.4
Teresa M. Chan	15 (0.6%)	15.7
King-Wa Fu	15 (0.6%)	9.9
Isaac Chun-Hai Fung	15 (0.6%)	9.9
Jenine K. Harris	15 (0.6%)	14.7
Michelle Lin	15 (0.6%)	18.8
Brent Thoma	15 (0.6%)	13.7
Michael A. Thompson	15 (0.6%)	12.5
Zion Tsz Ho Tse	15 (0.6%)	9.9
**Institutions**		
University of California System	143 (5.5%)	17.4
Harvard University	95 (3.7%)	15.6
University of Pennsylvania	67 (2.6%)	13.0
University of Texas System	63 (2.4%)	10.8
Johns Hopkins University	58 (2.2%)	18.0
University of London	58 (2.2%)	8.4
Pennsylvania Commonwealth System of Higher Education	57 (2.2%)	27.0
University of Toronto	57 (2.2%)	33.5
University System of Georgia	56 (2.2%)	10.2
University of North Carolina	49 (1.9%)	13.4
**Countries**		
United States	1344 (52.1%)	14.2
United Kingdom	314 (11.1%)	11.1
Australia	209 (8.1%)	14.0
Canada	203 (7.9%)	18.5
China	124 (4.8%)	8.8
Spain	104 (4.0%)	8.0
India	91 (3.5%)	2.8
Italy	66 (2.6%)	7.3
South Korea	57 (2.2%)	10.0
Saudi Arabia	54 (2.1%)	4.5
**Journals (2018 Impact factor, Quartile)**		
Journal of Medical Internet Research (4.945, Q1)	139 (5.4%)	26.0
PLoS ONE (2.776, Q2)	71 (2.8%)	35.0
Journal of Health Communication (1.773, Q2)	32 (1.2%)	11.2
Lecture Notes in Computer Science (NA)	31 (1.2%)	4.4
Studies in Health Technology and Informatics (NA)	30 (1.2%)	5.0
Computers in Human Behavior (4.306, Q1)	27 (1.0%)	19.3
Health Communication (1.846, Q2)	24 (0.9%)	10.3
International Journal of Environmental Research and Public Health (2.468, Q2)	22 (0.9%)	3.8
BMJ Open (2.376, Q2)	21 (0.8%)	9.2
American Journal of Infection Control (1.971, Q2)	15 (0.6%)	37.1
**Web of Science Categories**		
Public environmental occupational health	304 (11.8%)	13.5
Health care sciences services	301 (11.7%)	19.6
Computer science information systems	283 (11.0%)	7.9
Medical informatics	268 (10.4%)	17.9
Computer science theory methods	224 (8.7%)	5.8
Communication	164 (6.4%)	16.6
Computer science artificial intelligence	155 (6.0%)	8.9
Computer science interdisciplinary applications	142 (5.5%)	7.8
Information science library science	139 (5.4%)	8.2
Engineering electrical electronic	126 (4.9%)	4.3

*For journals belonging to multiple categories, the best impact factor quartile is listed*.

**Figure 3:**
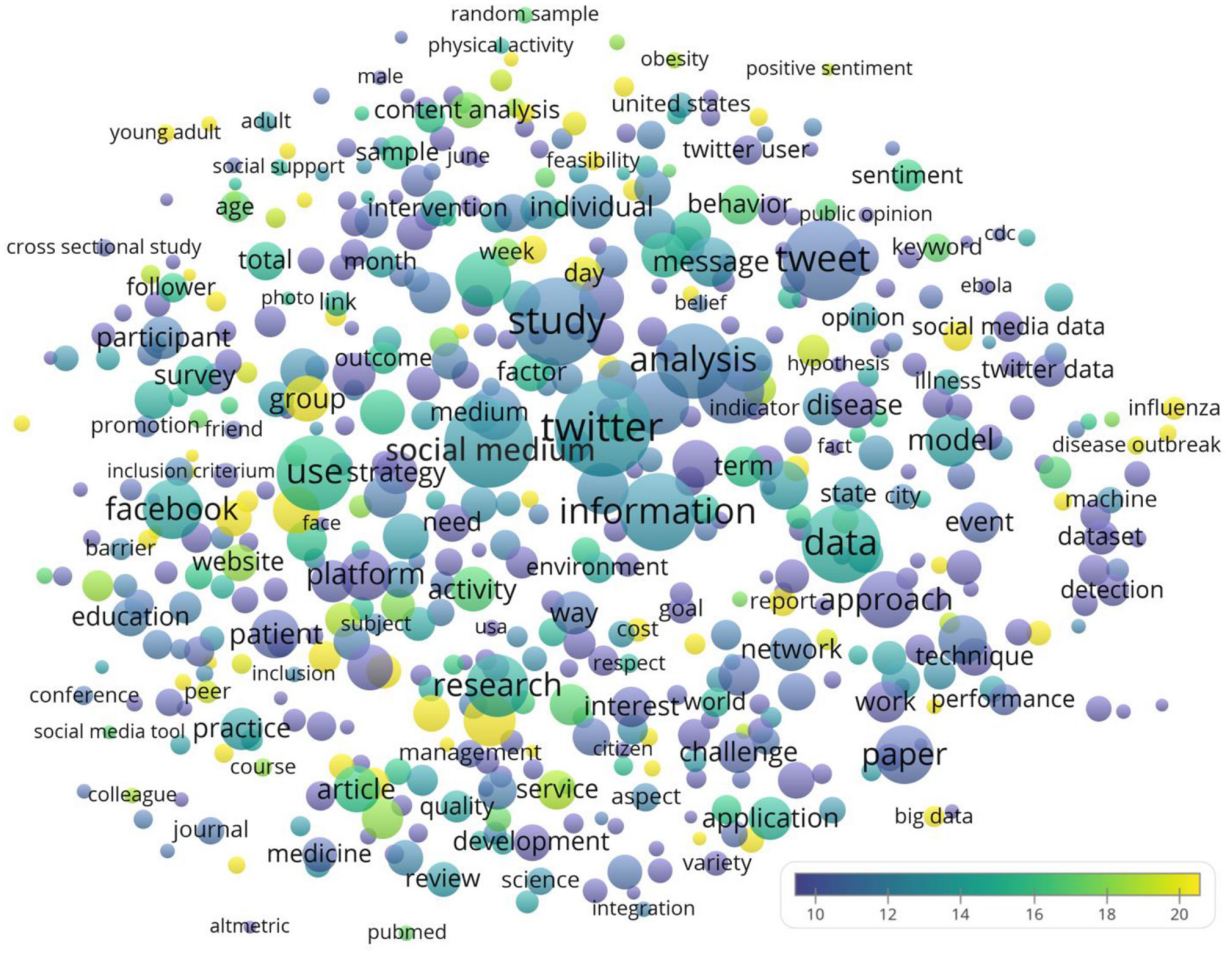
Term map showing the recurring terms mentioned in at least 1% (*n* = 26) of the titles and abstracts of the papers concerning Twitter and health. Bubble size indicated the number of papers mentioning the term. Bubble color indicated the citations per paper. The proximity between bubbles indicated how frequently the terms were mentioned in the same papers.

A term map presented in Figure 3 displays the terms mentioned in the titles and abstracts of the papers. Some of the more common terms included social medium (*n* = 1,184, CPP = 13.0), study (*n* = 1,153, CPP = 11.8), tweet (*n* = 966, CPP = 11.1), information (*n* = 915, CPP = 12.7) and analysis (*n* = 835, CPP = 11.7). The top 20 terms with highest CPP are listed in Table 2. Interestingly, flu was a frequently recurring term, with adolescent being a commonly mentioned age group.

**Table 2:** Top 20 terms with the highest citations per paper (CPP).

**Term**^**a**^	***n***	**CPP**
Online community	32 (1.2%)	63.2
Rise	44 (1.7%)	51.8
Culture	48 (1.9%)	39.6
Phenomenon	52 (2.0%)	39.6
Marketing	73 (2.8%)	38.9
Flu^b^	47 (1.8%)	36.7
Big data	71 (2.7%)	33.6
Cost	88 (3.4%)	33.5
Inclusion criterium	29 (1.1%)	32.7
Social media activity	28 (1.1%)	32.5
Adolescent	37 (1.4%)	32.2
Social networking site	77 (3.0%)	29.3
Social media site	63 (2.4%)	28.1
Real time	95 (3.7%)	27.4
Microblog	39 (1.5%)	27.1
Influenza^b^	81 (3.1%)	26.4
Citation	49 (1.9%)	26.3
Interaction	180 (7.0%)	25.5
Social	31 (1.2%)	25.5
Social networking	44 (1.7%)	25.2

a *Only terms that appeared in at least 1% of the papers were considered*.

b *The presence of the synonyms “Flu” and “Influenza” among the top 20 terms clearly indicates that this disease represents one of the most significant areas for Twitter-based medical research*.

**Figure 4:**
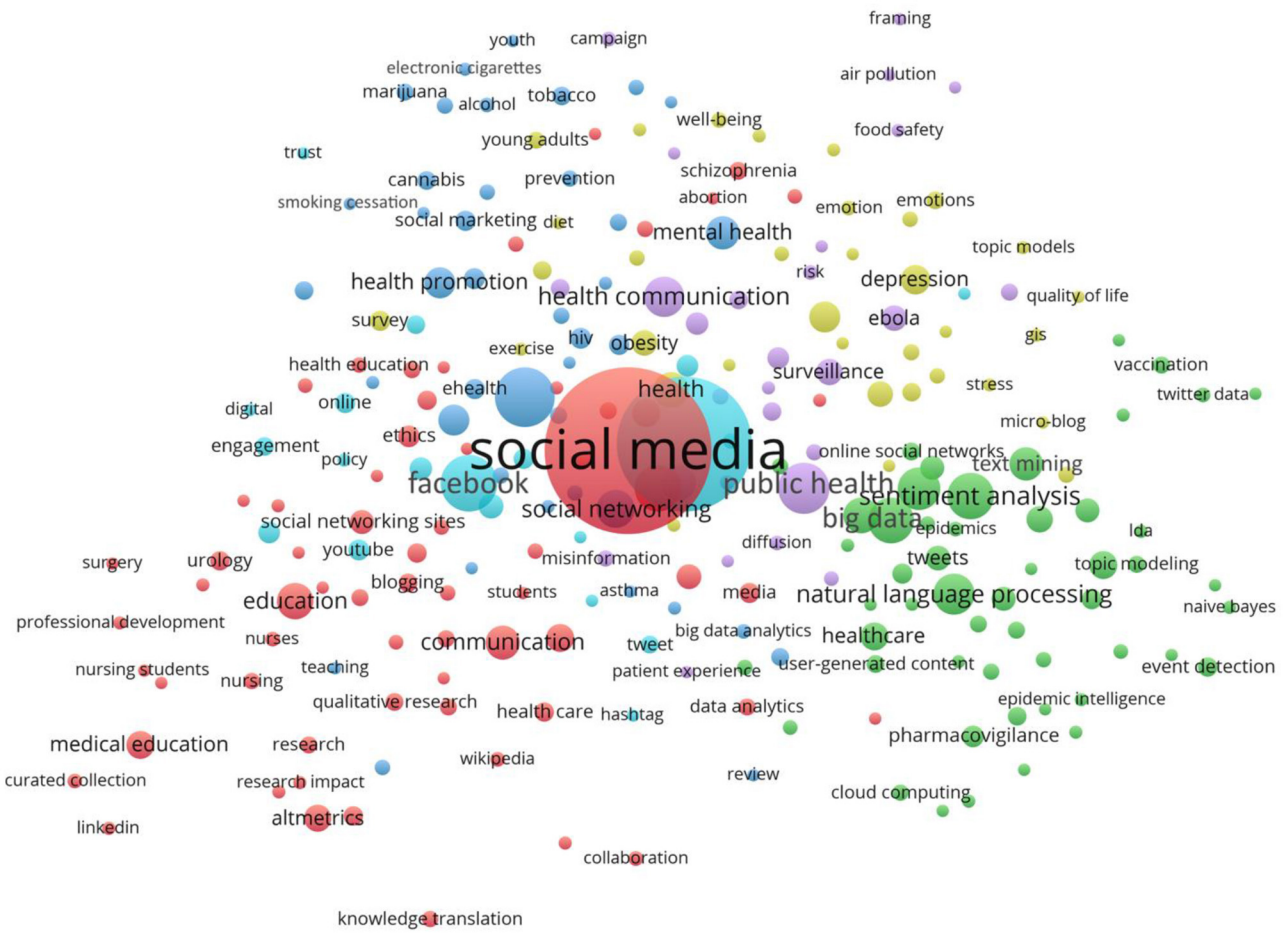
Keyword map showing the recurring author keywords (at least *n* = 5) from the papers concerning Twitter and health. Bubble size indicated the number of papers mentioning the term. Bubble color indicated the clustering. The default parameters of VOSviewer were used and the minimal cluster size was set as 20. There were 66 keywords in cluster 1 (red) related to professional education in healthcare sector; 52 words in cluster 2 (green) related to the big data and sentiment analysis; 41 words in cluster 3 (blue) related to the social marketing and substance use; 34 words in cluster 4 (yellow) related to the physical and emotional well-being of young adults; 28 words in cluster 5 (purple) related to public health and health communication; 21 words in cluster 6 (indigo) related to various social media such as Facebook and YouTube. The proximity between bubbles indicated how frequently the terms were mentioned in the same papers.

A keyword map is shown in Figure 4. The keyword map (Figure 4) displays the six identified clusters relating to various recurring themes. The largest cluster consisted of 66 keywords and was related to professional education in healthcare. The top 20 most cited keywords are listed in Table 3. The recurring themes seemed to relate to cyberbullying, medicine 2.0, ethics, and population surveillance.

**Table 3:** Top 20 author keywords with the highest citations per paper (CPP).

**Keyword**	***n***	**CPP**
Epistemology	2 (0.1%)	806.0
Analytics	3 (0.1%)	544.7
Social network sites	4 (0.2%)	460.0
Facebook depression	2 (0.1%)	277.5
Bullying	2 (0.1%)	275.0
Online harassment	2 (0.1%)	273.0
Power law	2 (0.1%)	229.5
Scientometrics	2 (0.1%)	229.0
Children	3 (0.1%)	187.7
Antibiotic	2 (0.1%)	158.0
Publishing	3 (0.1%)	156.7
Information storage and retrieval	2 (0.1%)	148.0
Cyberbullying	4 (0.2%)	147.0
Medicine 2.0	4 (0.2%)	124.8
Ethics	16 (0.6%)	114.8
Population surveillance	3 (0.1%)	106.3
Teaching	5 (0.2%)	103.6
Semantic web	2 (0.1%)	101.5
Biosurveillance	3 (0.1%)	100.7
Forecasting	3 (0.1%)	98.7

*Only keywords that appeared in at least 2 of the papers were considered*.

## Discussion

The analysis was based on 2,582 papers, of which the majority was original articles. The number of Twitter based papers published in each year increased over the period. More people are using Twitter as a channel and data source to do research, because it is one of the most popular forms of social media used for healthcare communication (24). For instance, its data has been entered into machine learning models for content classification, which well-demonstrated the potential of Twitter as a source for the collection, storage, visualization, and analysis of healthcare-related Big Data in real time, and allowed assessment of relevant parameters such as health activity and nutritional habits (25, 26).

Based on author keywords, several clusters of themes were identified by VOSviewer (with reference to Figure 4 as seen in different colors). Two authors (AWKY and AGA) examined the data to see which words were recurring in each cluster and thus defined the framework for the following discussion of the themes. The diversity of identified themes ranges from professional education in healthcare, to big data and sentiment analysis, social marketing and substance use, physical and emotional well-being of young adults, public health and health communication, and use of various other social media platforms such as Facebook and YouTube.

Regarding professional education in healthcare, several advantages of Twitter as a medium for exchange of knowledge were proposed, such as connection of practice communities, development of scholarly work via crowdsourcing, distribution of most recent information, acceleration of knowledge translation and post-publication peer review, engaging the public, and building a support network (27). For instance, the use of Twitter by postgraduate pharmacy students during class was deemed to facilitate sharing of ideas among the class, with over 80% of students participating, indicating that this encouraged them to express their opinion when they would not have done otherwise (28). However, Twitter contains high volumes of information which might cause information overload, distraction, and a propagation of wrong information (27). Therefore, it was advised that an authority should convey credible information sources to the professional community and ground rules for the use of Twitter should be set for students and incorporated in class activities. Example of the latter is usage of Twitter for real-time discussions and informal quizzes and polls for a predefined period (29). Besides pharmacy, the use of Twitter was also incorporated into the education of anatomy (30), nephrology (31) nursing (32), and other medical specialties.

The abundance of information contained in Twitter enabled many health-related analyses and predictions with big data and sentiment analysis. For example, by analyzing the language expressed in different tweets it was possible to predict mortality due to atherosclerotic heart disease in different communities. In particular, tweets expressing anger, negative relationships or emotions, disengagement and anxiety were positively correlated to mortality (33). Interestingly, information in this tweets was found to be a better predictor of mortality than classical risk factors, such as smoking, diabetes and obesity (33). In another study, the number of asthma-related tweets was found to predict the number of asthma-related emergency department visits (34). However, readers should be aware that the prediction performance might vary depending on use of different statistical models.

In terms of social marketing and substance use, Twitter was utilized as a platform to market various products, such as alcoholic beverages, with at least one tweet per week to one tweet per day, thereby generating hundreds to thousands of product-related tweets (35, 36). Similar marketing on Twitter was done for electronic cigarettes (37) and hookah pipes (38). Along the same line, the majority of tweets concerning marijuana and cannabis edibles were rated to be positive toward their usage (39, 40). One potential pitfall of social media is a relatively low level of content regulation, as demonstrated in a study were a fictitious advertisement for illicit online drug sales was distributed on Twitter and other social media platforms and remained accessible for months (41).

In the context of physical and emotional well-being of young adults, social media might inadvertently act as a platform for cyberbullying leading to depression and anxiety (42). Obesity was one of the most common topics triggering tweets with emotionally evocative and humorous content, whereby especially tweets containing derogatory jokes, were more frequently retweeted (43). Another relevant phenomenon is that some adolescents might develop symptoms of depression once they get offline after a prolonged period of immersing in online activities (42). Overall, the use of multiple social media platforms, including Twitter, was associated with increased levels of depression and anxiety (44). Furthermore, the time used on social media was positively associated with a perceived social isolation score (45).

Concerning public health and health communication, surveillance was one of the largest topics. Twitter and other social media could be successfully used to track disease activity and public concern during the Influenza A H1N1 outbreak in 2009 in the United States (46), the cholera outbreak in Haiti in 2010 (22), the worldwide Ebola outbreak in 2014 (47) and the worldwide COVID-19 outbreak in 2019–2020 (48). Public health surveillance via Twitter was similarly done for themes not related to outbreaks of infectious disease such as dental pain (49). Public health topics also included campaigns launched on Twitter and other social media platforms to promote food safety (50), awareness for cervical cancer (51), and prevention of adolescent dating abuse (52). We noticed that whereas public health and medicine were frequently investigated subjects, the WoS journal category of dentistry had only 16 papers. This apparently formed a research gap to be filled in future studies.

Many of the examples stated above also illustrate that social media platforms are often investigated together, as social networking involves a multitude of different platforms (e.g., Twitter, Facebook, YouTube, Instagram, Reddit, Snapchat). A large amount of information is continuously circulating on Twitter reaching a broad audience and reflecting on different health-related issues worldwide. With the worldwide reach and data availability characteristic of Twitter, the analyzed literature set on Twitter and health had a considerable number of contributions from Africa and the Middle East, such as Saudi Arabia (2.1%) and South Africa (0.6%), whereby contributions from Africa were limited on public health related research in general (53).

## Limitations

Due to the involvement of citation counts, a single database, WoS, was selected to extract the data. Therefore, possible publications not indexed in this database are missing from this analysis. Some identified terms and keywords might be synonyms for others. However, merging such terms and keywords would not be appropriate since it would represent manipulation of original data and therefore might distort the validity of outcomes. Moreover, WoS mainly indexed papers written in English, so non-English literature was scarcely covered. Along this line, readers should also be aware that some countries may have their own alternatives to Twitter, such as Weibo in China, which was not covered in this study. On the other hand, readers should be aware that citation count does not directly reflect the quality of the cited work, and that citation count could be inflated by self-citation. Therefore, this work assessed CPPs of terms and entities from various levels, instead of the citation count of individual works.

## Conclusions

This bibliometric analysis based on 2,582 papers concerning Twitter and health shows that the majority are original articles with worldwide contributions. These papers often investigated Twitter together with other social media platforms, such as YouTube and Facebook. We identified a high diversity of themes ranging from professional education in healthcare, to big data and sentiment analysis, social marketing and substance use, physical and emotional well-being of young adults, and public health and health communication. This diversity of themes and approaches warrants further broad and versatile use of Twitter for health-related research. The recurring contributors, journals and research themes reported in this study may be useful for researchers to identify potential collaborations and research directions.

## Data Availability Statement

The original contributions presented in the study are included in the article/supplementary material, further inquiries can be directed to the corresponding authors.

## Author Contributions

AA and AY: conceived and designed the study and drafted the initial manuscript draft. AY: extracted and analyzed the data. All authors critically revised the manuscript, interpreted data, and approved the final manuscript. All authors contributed to the article and approved the submitted version.

## Conflict of Interest

The authors declare that the research was conducted in the absence of any commercial or financial relationships that could be construed as a potential conflict of interest.
